# Inhaling Hydrogen Ameliorates Early Postresuscitation EEG Characteristics in an Asphyxial Cardiac Arrest Rat Model

**DOI:** 10.1155/2019/6410159

**Published:** 2019-10-16

**Authors:** Gang Chen, Jingru Li, Jianjie Wang, Bihua Chen, Yongqin Li

**Affiliations:** ^1^Department of Biomedical Engineering, Army Medical University, Chongqing 400038, China; ^2^The Centre for Disease Control and Prevention of Southern War Zone, Yunnan Province, Kunming 650000, China

## Abstract

**Background:**

Electroencephalography (EEG) is commonly used to assess the neurological prognosis of comatose patients after cardiac arrest (CA). However, the early prognostic accuracy of EEG may be affected by postresuscitation interventions. Recent animal studies found that hydrogen inhalation after CA greatly improved neurological outcomes by selectively neutralizing highly reactive oxidants, but the effect of hydrogen inhalation on EEG recovery and its prognostication value are still unclear. The present study investigated the effects of hydrogen inhalation on early postresuscitation EEG characteristics in an asphyxial CA rat model.

**Methods:**

Cardiopulmonary resuscitation was initiated after 5 min of untreated CA in 40 adult female Sprague-Dawley rats. Animals were randomized for ventilation with 98% oxygen plus 2% hydrogen (H2) or 98% oxygen plus 2% nitrogen (Ctrl) under normothermia for 1 h. EEG characteristics were continuously recorded for 4 h, and the relationships between quantitative EEG characteristics and 96 h neurological outcomes were investigated.

**Results:**

No differences in baseline and resuscitation data were observed between groups, but the survival rate was significantly higher in the H2 group than in the Ctrl group (90% vs. 40%, *P* < 0.01). Compared to the Ctrl group, the H2 group showed a shorter burst onset time (21.85 [20.00–23.38] vs. 25.70 [22.48–30.05], *P* < 0.01) and time to normal trace (169.83 [161.63–208.55] vs. 208.39 [186.29–248.80], *P* < 0.01). Additionally, the burst suppression ratio (0.66 ± 0.09 vs. 0.52 ± 0.17, *P* < 0.01) and weighted‐permutation entropy (0.47 ± 0.16 vs. 0.34 ± 0.13, *P* < 0.01) were markedly higher in the H2 group. The areas under the receiver operating characteristic curves for the 4 EEG characteristics in predicting survival were 0.82, 0.84, 0.88, and 0.83, respectively.

**Conclusions:**

In this asphyxial CA rat model, the improved postresuscitation EEG characteristics for animals treated with hydrogen are correlated with the better 96 h neurological outcome and predicted survival.

## 1. Introduction

With morbidity ranging from 35 to 125 per 100,000 persons, cardiac arrest (CA) remains a major public health issue and the most common cause of death worldwide [1]. Despite decades of effort to improve outcomes following CA, only 10.6% of adults who experienced out-of-hospital CA and 23.8% of adults who experienced in-hospital CA survived in 2015 [2]. Two-thirds of patients who achieved restoration of spontaneous circulation (ROSC) died within days due to significant neurologic disability and myocardial dysfunction, and fewer than 30% of survivors returned to a normal functional lifestyle [3]. Early recognition of patients without a chance of recovering brain function might prevent the continuation of futile treatment and improve communication between doctors and patients. Moreover, an early, reliable prognosis for postresuscitation comatose patients could help caregivers make sensible decisions regarding the intensity of care [4].

Electroencephalography (EEG) activity mainly reflects cortical synaptic activity [5] and is widely used to assess neurological prognosis in comatose patients after CA [6]. Persistent isoelectricity, low voltage activity, or burst-suppression patterns in EEG activity in the first 24 h predict a poor outcome without false positives, while rapid recovery towards continuous patterns within 12 h is strongly associated with a good neurological outcome [7]. The evolution of EEG patterns over time can be displayed by quantitative EEG analysis, and quantitative EEG features such as the burst suppression ratio (BSR), response entropy, state entropy, and sub-band entropy differ between good- and poor-outcome groups during the first 24 h after CA [8]. Although the EEG test is a part of the current prognostication guidelines, a meta-analysis of EEG indicated that the provision of accurate recommendations is limited by the influence of confounding factors such as sedation, neuromuscular blockade, and targeted temperature management, which might lead to overestimation of the specificity of the results [9].

The neuroprotective effects of postresuscitation hydrogen inhalation have been evaluated in many studies. Hayashida et al. found that inhalation of hydrogen gas was a favourable strategy to mitigate mortality and improve functional outcomes of post-CA syndrome in a ventricular fibrillation rat model [10]. Hu et al. observed that hydrogen-rich saline treatment improved survival and neurological outcome after CA/resuscitation in rats [11]. Our previous studies also indicated the neuroprotective effects of hydrogen inhalation were superior to targeted temperature management in rats [12, 13]. After ischemia/reperfusion, reactive oxygen species is massively produced in the brain and the oxidative damage to brain tissues has been regarded as a fundamental mechanism of postresuscitation brain injury. The neuroprotective role of hydrogen is primarily through selective reactive oxygen species attenuation [10–13]. However, the effect of hydrogen inhalation on characteristics of early postresuscitation EEG and its prognostication value are still unclear. In the current study, we investigated the effects of hydrogen inhalation on the quantitative characteristics of early postresuscitation EEG measurements in a CA rat model.

## 2. Materials and Methods

### 2.1. Animal Preparation

All procedures of this prospective, randomized animal studywere approved by the animal investigation ethics committee of Army Medical University. According to the National Guide for the Care and Use of Laboratory Animals, all animals received humane care throughout the experiment period.

Forty adult female Sprague-Dawley rats (10–12 weeks age) weighing 208–289 g were provided by the Laboratory Animal Centre of Army Medical University and used in this study. All animals were fasted overnight, with free access to water. A dose of 45 mg/kg pentobarbital sodium was injected via intramuscular route to initiate anaesthesia. Additional doses of 10 mg/kg pentobarbital sodium were administered to maintain anaesthesia. After confirming anaesthesia, the animal was fastened on a surgical board in the supine position, and a 14-gauge cannula (Abbocath-T, Abbott Laboratories, North Chicago, IL, USA) was intubatedorally into the trachea for mechanical ventilation. Mechanical ventilation was controlled by a volume-controlled ventilator (ALC-V9, Alcott Biotech CO. Ltd, Shanghai, China) with a tidal volume of 6.5 ml/kg and a FiO2 of 0.21. To measure arterial pressure and acquire a blood sample, a polyethylene tubing PE-50 (Instech Laboratories Inc., Plymouth Meeting, PA, USA) was inserted into the right femoral arteryand advanced to the thoracic/descending aorta to allow the administration of fluids and drugs, the left femoral vein was cannulated with another PE-50 catheter. A multiparameter monitor (Model 90369, Spacelabs, Snoqualmie, WA, USA) was used to continuously evaluate the arterial pressure and conventional lead II ECG. To ensure normothermia (36.5 –37.5°C), a heat lamp was operated following the core temperature monitored by a thermocouple sensor (TH212, Bjhocy Science and Technology Co. Ltd., Beijing, China) that was placed into the esophagus. A couple of subdermal needles were inserted into both left and right surfaces of the skull respectively to record the EEG signals [14]. To prevent clotting in catheters, they were flushed intermittently with saline containing 2.5 IU/ml crystalline bovine heparin.

### 2.2. Induction of CA

The CA was induced as previously descripted [14]. Briefly, after collection of baseline data and removal of the thermocouple sensor, the mechanical ventilator was turned off, and the endotracheal tube was clamped to induce asphyxia. The mean arterial pressure (MAP) of less than 20 mmHg was considered as a successful induction of CA whatever the cardiac rhythm was pulseless electrical activity or asystolic. In our experiment, CA occurred approximately 3 min after initiation of asphyxia.

### 2.3. Cardiopulmonary Resuscitation

Cardiopulmonary resuscitation (CPR) was performed as previously descripted [12], with the following modification. CPR, including chest compression and ventilation, was initiated after 5 min of untreated CA. Chest compressions were delivered by a finger of the same experienced investigator at a compression rate of 200/min with a depth of 25–30% of the anterior posterior diameter of the animal's chest. At the beginning of chest compression, the animals were ventilated at 80 breaths/minute with tidal volume of 6.5 ml/kg and FiO_2_ of 1.0. One minute after the start of CPR, a dose of epinephrine (0.02 mg/kg) was given and repeated at 3-minute intervals as needed [10, 15]. ROSC was defined as an organized cardiac rhythm with the MAP at or greater than 60 mmHg for an interval exceeding 5 min [14]. CPR was stopped when ROSC was observed or the duration of CPR was over 10 min.

### 2.4. Postresuscitation Care

Immediately following ROSC, animals were assigned to the H2 group or Ctrl group (each *n* = 20) according to random number table method. In H2 group, animals were ventilated with 2% hydrogen plus 98% oxygen. In Ctrl group, animals were ventilated with 2% nitrogen plus 98% oxygen. Animals in both groups received intensive care for an additional 4 hours after ROSC under normothermia (36.5–37.5℃). Animals in the experimental group were ventilated mechanically for 1 hour with a mixture of 2% hydrogen and 98% oxygen, then for 3 hours with air, while animals in the control group were ventilated for 1 hour with a mixture of 2% nitrogen and 98% oxygen, and then for 3 hours with air. Four hours after resuscitation, all implanted experimental devices, including catheters, sensors, and hypodermic needles, were removed and the wound was surgically sutured. Animals were then returned to their cages, wherethe temperature was maintained at 20.0–25.0℃, and observed for up to 96 h.

### 2.5. Measurements

EEG was continuously performed for all animals for the first 4 h after ROSC. The raw EEG signal was amplified and conditioned by a two-channel differential preamplifier (PRE-ISO. EEG100, Xiangyun Computing Technology, Beijing, China). The amplified EEG was recorded through a data acquisition system (Windaq hardware/software, Dataq Instruments Inc., Akron, OH, USA) at a sample rate of 1000 Hz.

Arterial blood samples were collected at baseline and at 2 and 4 h after ROSC. Blood gases were measured with the aid of a blood analyser (i15, Edan Instruments Inc, Shenzhen, China). The blood sample was stored at 4°C overnight to clot and then was centrifuged, and the serum was extracted and stored in −80°C. The concentration of the astroglial protein S100 beta (S100B) in the serum of arterial blood samples was quantified with an enzyme-linked immunoassay (Elisa Kit, Cusbio Biotech Co Ltd, Wuhan, China) and served as a biomarker of cerebral injury [16, 17].

The neurological deficit score (NDS) was performed daily for 96 h postresuscitation by two investigators blinded to the treatment. The NDS was used to evaluate neurological outcome after global cerebral ischemia for rat. NDS evaluates consciousness, breathing, cranial nerve reflexes, motor function, sensory function, and coordination with score from 0 to 500 scale (0: no observed neurological deficit, 500: death or brain death) [18]. After the last evaluation of NDS, at 96 h postresuscitation, surviving animals were euthanized by a lethal intraperitoneal injection of sodium pentobarbital (150 mg/kg) [19].

### 2.6. Quantitative Analysis of EEG Measurements

After the experiment was finished, three patterns, including isoelectric/suppression, burst suppression, and continuous background, were annotated in the 4 h EEG recording of each animal by two investigators who were blinded to the treatment [20]. When there was no visible EEG activity for more than 30 sec in the EEG recording, it was annotated as isoelectric/suppression. When the EEG recording turned to obvious increases in amplitude (amplitude >10 *μ*V in both the left and right channels) followed by no EEG activity or low amplitude activity of at least 0.5 sec (amplitude <10 *μ*V in both the left and right channels), it was annotated as burst suppression. The continuous background pattern was identified when the maximum amplitude of recording >10 *μ*V meanwhile the minimum amplitude >5 *μ*V [21]. After the classification of early postresuscitation EEG recording, the onset time of burst (OTOB) was defined as the time from ROSC to the initial burst suppression pattern, and the time to normal trace (TTNT) was defined as the time from ROSC to the initial continuous background pattern [14].

A 1 min artefact-free segment of raw EEG was extracted at baseline, 5 min postresuscitation and every 30 min after ROSC. Then, all EEG segments were pre-processed using a bandpass filter with cut-off frequencies of 0.3–100 Hz to remove offset and high-frequency noise; 50 Hz notch filtering was performed to remove the effects of power frequency interference [22]. For each segment, the BSR was defined as the ratio of the duration of the burst signal to the duration of signal [23]. The weighted-permutation entropy (WPE) was calculated to describe the complexity and irregularity of EEG recordings using a previously established algorithm [24]. The BSR and WPE were compared between the two groups at all time points. To evaluate the overall predictive ability of BSR and WPE, we calculated the time-weighted average (TWA) values for the two features in a manner of calculating epidemiological exposures over time [25, 26], and named them TWA-BSR and TWA-WPE. Then the predictive power of OTOB, TTNT, TWA-BSR, and TWA-WPE was subsequently determined.

### 2.7. Statistical Analysis

Statistical analysis was performed using IBM SPSS statistic 22.0 software (Chicago, IL, USA). Normally distributed quantitative variables were confirmed by Kolmogorov–Smirnov test and reported as the mean ± standard deviation. For nonnormally distributed quantitative variables, we used the median [first quartile–third quartile]. Categorical data were summarized by ratios or percentages. For data with normal distribution and homogeneity of variance, a two-tailed Student's *t* test was used, while nonparametric test was used for data with nonnormal distribution or heterogeneity of variance. Kaplan–Meier analysis was performed to obtain Survival curves and survival time was compared between the two groups with a log-rank test. The relationships between EEG characteristics and the S100B value, and the 96 h NDS were analyzed using Spearman's correlation. The predictive power of EEG characteristics was assessed by the area under the receiver operating characteristic curve (ROC). A value of *P* < 0.05 was considered statistically significant.

## 3. Results

### 3.1. Baseline and Resuscitation

There were no significant differences in body weight, heart rate, MAP, or core temperature between the H2 and Ctrl groups at baseline. CA was successfully induced in all animals in the two groups, and no significant differences were observed in the asphyxial time required to induce CA between the H2 and Ctrl groups. There were no significant differences in the period of CPR, total dosage of epinephrine, and resuscitation rate (Table 1).

### 3.2. Neurological Outcome and Survival

As shown in Table 2, the NDS measurements during the 96 h observational period were significantly higher in the Ctrl group than in the H2 group.

**Figure 1:**
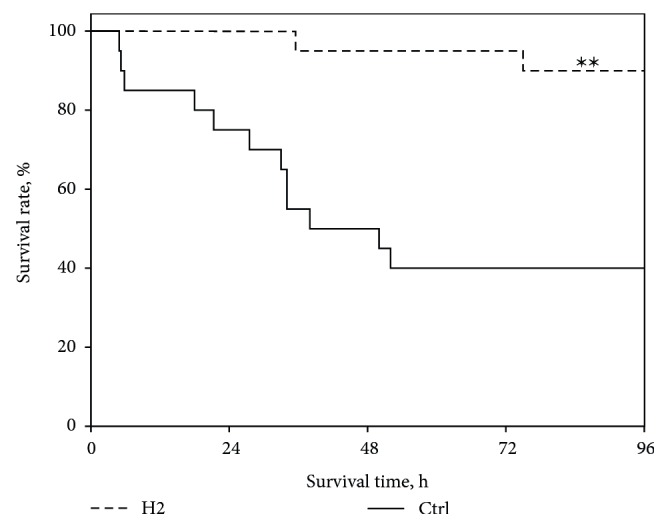
Kaplan-Meier analysis of cumulative survival at 96 h postresuscitation; *n* = 20 in each group. Ctrl, control group; H2, hydrogen inhalation group. ∗∗*P* < 0.01 compared with the control group.

The survival rate was significantly higher in the H2 group (90% vs. 40% *P* < 0.01) than in the Ctrl group at 96 h after ROSC. The survival time (44.00 h [22.85–96.00] vs. 96.00 h [96.00–96.00], *P* < 0.01) was shorter and the cumulative survival rate was significantly lower in the Ctrl group than in the H2 group (Figure 1).

### 3.3. Postresuscitation Brain Injury and Blood Gas Change

**Figure 2:**
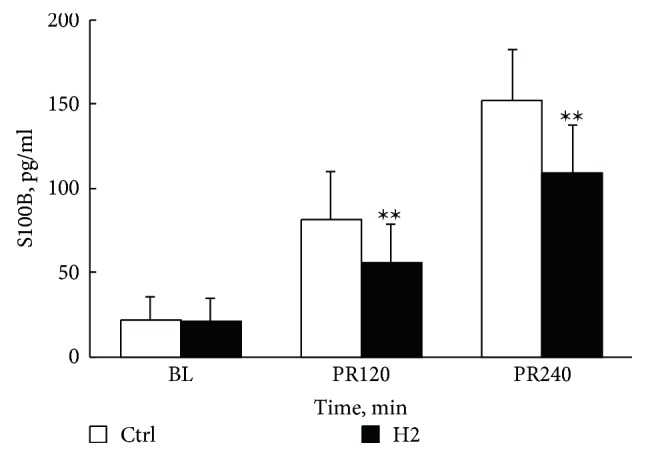
Serum level S100B measured at baseline, 120 and 240 min after resuscitation; *n* = 20 at each time point in two groups. Ctrl, control group; H2, hydrogen inhalation group; BL, baseline; PR, postresuscitation. ∗∗*P* < 0.01 compared with the Control group.

As shown in Figure2, pre-CA serum S100B levels did not differ significantly between groups. Compared with those in the Ctrl group, serum S100B levels in the H2 group were greatly suppressed, both at 120 min and at 240 min after resuscitation. However, there were no significant differences in arterial gas measurements between Ctrl and H2 groups at any time point (Table 3).

### 3.4. Differences in the Quantitative EEG Characteristics

**Figure 3:**
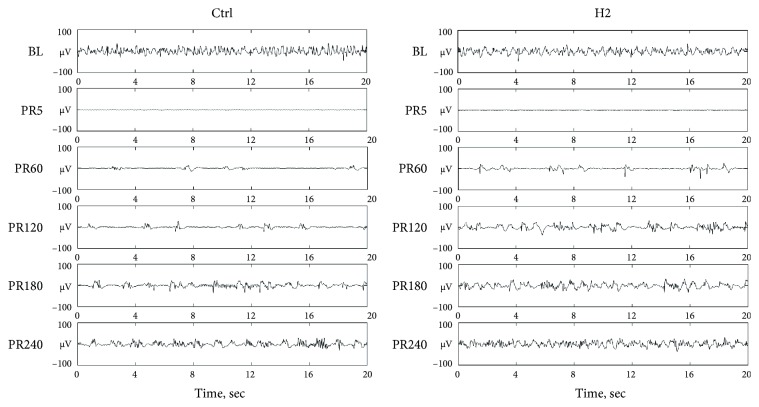
Representative EEG patterns for the control group and hydrogen inhalation group. Ctrl, control group; H2, hydrogen inhalation group; BL, baseline; PR, postresuscitation.

In the 4 h postresuscitation phase after ROSC, three EEG recovery patterns, including isoelectric tracing, burst suppression, and continuous background EEG activity, were observed in all animals (Figure 3), except three rats that remained in burst-suppression patterns at 4 h after resuscitation.

**Figure 4:**
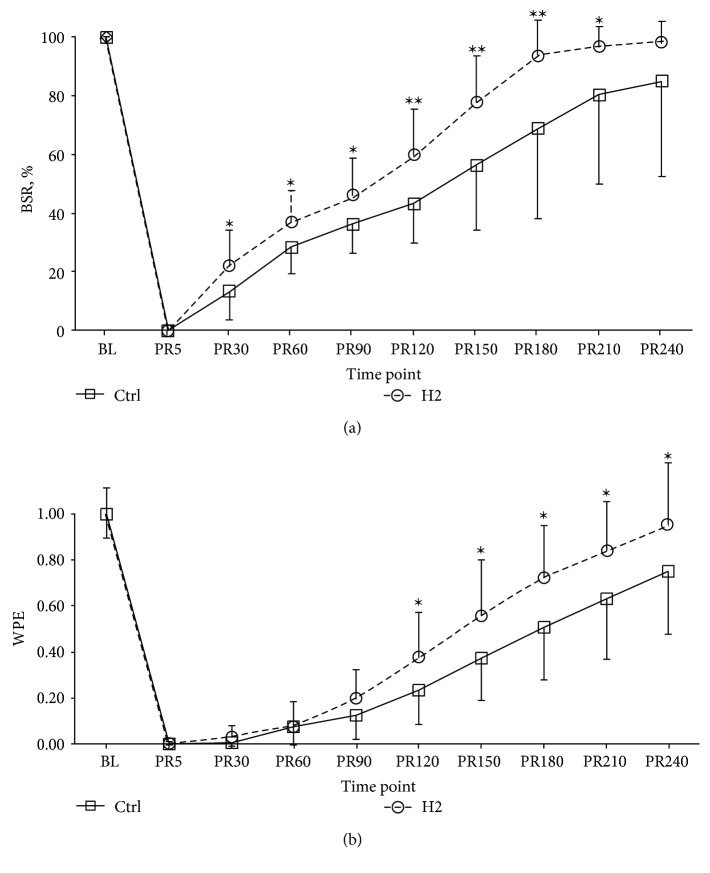
The BSR (a) and WPE (b) at baseline and during the first 4 h after resuscitation; *n* = 20 at each time point in two groups. BL, baseline; PR, postresuscitation; BSR, burst suppression ratio; WPE, weighted-permutation entropy. ∗ *P* < 0.05 and ∗∗*P* < 0.01 compared with the control group.

Compared to those in the Ctrl groups, the OTOB (21.85 [20.00–23.38] vs. 25.70 [22.48–30.05], *P* < 0.01) and TTNT (169.83 [161.63–208.55] vs. 208.39 [186.29–248.80], *P* < 0.01) in the H2 group were significantly shorter.  Figure 4 illustrates the temporal evolution of the BSR and WPE at baseline and during the 4-h postresuscitation period between the Ctrl and H2 groups.

The BSR in the H2 group was significantly higher than that in the Ctrl group from 0.5 h to 3.5 h postresuscitation, while the WPE was higher from 2 h to 4 h. Both the TWA-BSR (0.66 ± 0.09 vs. 0.52 ± 0.17, *P* < 0.01) and TWA-WPE (0.47 ± 0.16 vs. 0.34 ± 0.13, *P* < 0.01) were significantly higher in the H2 group than in the Ctrl group.

### 3.5. Predictive Value of Quantitative EEG Characteristics

**Figure 5:**
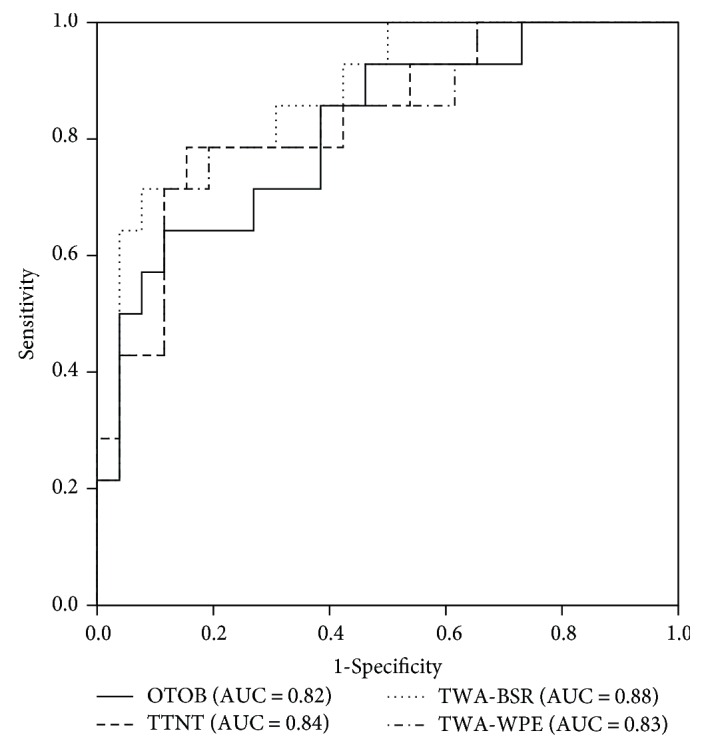
Receiver operating characteristic curves and area under the curves for the onset time of burst, time to normal trace, time-weighted average burst suppression ratio, and time-weighted average weighted-permutation entropy. OTOB, onset time of burst; TTNT, time to normal trace; BSR, burst suppression ratio; WPE, weighted-permutation entropy; TWA, time-weight average.

Spearman's correlation showed that the OTOB (*r* = 0.68, 95% CI, [0.47, 0.82], *P* < 0.01), TTNT (*r* = 0.84, 95% CI, [0.72, 0.91], *P* < 0.01), TWA-BSR (*r* = −0.92, 95% CI, [−0.96, −0.82], *P* < 0.01), and TWA-WPE (*r* = −0.90, 95% CI, [−0.95, −0.80], *P* < 0.01) were correlated with postresuscitation serum S100B level. Correlation analysis also demonstrated that the OTOB (*r* = 0.56, 95% CI, [0.28, 0.77], *P* < 0.01), TTNT (*r* = 0.63, 95% CI, [0.40, 0.80], *P* < 0.01), TWA-BSR (*r* = −0.71, 95% CI, [−0.851, −0.51], *P* < 0.01), and TWA‐WPE (*r* = −0.69, 95% CI, [−0.84, −0.46], *P* < 0.01) were associated with the 96 h NDS. ROC curves showed that the OTOB (area under the ROC = 0.82, 95% CI, [0.68, 0.96], *P* < 0.01), TTNT (area under the ROC = 0.84, 95% CI, [0.70, 0.97], *P* < 0.01), TWA-BSR (area under the ROC = 0.88, 95% CI, [0.77, 0.99], *P* < 0.01), and TWA-WPE (area under the ROC = 0.83, 95% CI, [0.69, 0.96], *P* < 0.01) predicted survival at 96 h (Figure 5). When analyzed separately, the area under the ROC for OTOB, TTNT, TWA-BSR, and TWA-WPE was 0.76 (95% CI, [0.55, 0.98], *P* = 0.05), 0.88 (95% CI, [0.71, 1.00], *P* < 0.01), 0.84 (95% CI, [0.67, 1.00], *P* = 0.01), and 0.83 (95% CI, [0.64, 1.00], *P* = 0.01) in the Ctrl group and did not have significant difference compared with the overall results. However, the predictive capacity of these EEG characteristics could not be reliably assessed in the H2 group because of unbalanced survival data.

## 4. Discussion

To our knowledge, the present study was the first to demonstrate the effects of hydrogen inhalation on EEG parameters at early postresuscitation after CA. The quantitative EEG characteristics (OTOB, TTNT, BSR, and WPE) were significantly improved by hydrogen inhalation. The quantitative EEG characteristics were associated with the 96 h NDS and predicted survival.

Hydrogen inhalation has been confirmed as a potential therapeutic for neuroprotection after CA in previous studies. Huang et al. observed that molecular hydrogen can reduce cerebral ischemia-reperfusion injury and improve the prognosis of cardiopulmonary cerebral resuscitation [27]. Tomoyoshi et al. examined the feasibility and safety of hydrogen gas inhalation for human patients with post-CA syndrome [28]. The findings of the present study were consistent with those of previous studies and showed the efficacy of hydrogen inhalation. In this study, the NDS and 96 h survival rate were improved by inhaling 2% hydrogen for 1 h immediately after resuscitation. During recovery, the serum level of S100B also showed that brain injury was alleviated by hydrogen inhalation. These results reaffirmed the beneficial effect of hydrogen on neurological function and survival.

EEG primarily shows the sum of synchronous inhibitory and excitatory postsynaptic potentials. The EEG is also modulated and synchronized by subcortical “pacemaker neurons” in the thalamus and brainstem systems [29]. Therefore, EEG allowed the assessment of cortical and, to some extent, subcortical function. Because cortical synaptic activity is very sensitive to the effects of hypoxia-ischemia, the EEG results became isoelectric within ten to forty sec after circulatory arrest, which reflected massive cortical synaptic arrest [7]. As the mechanisms of postischemic brain injury unfold over hours to days following the re-establishment of adequate systemic circulation, the neurological function is also restored gradually, and the electrophysiological recovery of cortical activity can be monitored with EEG [30]. In the hours to days after ROSC, EEG showed the following sequential recovery phases: the isoelectric EEG phase, burst suppression phase and continuous cortical activity phase, as outlined by Jorgensen et al. [31]. The mechanisms of brain injury triggered by CA and resuscitation are complex and not fully understood [32]. However, previous studies have demonstrated that a brief episode of global brain ischemia produces selective and often extensive neuronal loss in vulnerable brain structures, such as the hippocampal CA1 pyramidal neurons [33]. Detrimental reactive oxygen species were found to be massively produced in the brain after ischemia-reperfusion, and oxidative damage to brain tissues is regarded as a fundamental mechanism of brain injury [33]. Reactive oxygen species, such as hydroxyl radicals and peroxynitrite, can be effectively scavenged by hydrogen molecules [34]. In addition, hydrogen was found to neutralize reactive oxygen species selectively, diffuse through bio-membranes and upregulate antioxidant enzyme gene expression in recent studies [35]. With hydrogen treatment, select vulnerable neuron subpopulations in the hippocampus, cortex, cerebellum, corpus striatum, and thalamus are conserved, delaying death [36]. Hayashida et al. also demonstrated that hydrogen inhalation rescued neuronal death and suppressed microglial activation in the hippocampus and cerebral cortex [37]. Thus, EEG parameters should also be improved at early postresuscitation since they directly reflect cortical and subcortical function.

However, reliable prognostications of CA outcome by EEG are confounded by the influence of hypothermia, sedation, and paralysis [30]. In a study by Jia et al., EEG burst frequency was unable to predict the outcome in patients with CA undergoing targeted temperature management [38]. Laurens et al. found that the value of EEG patterns in predicting outcome was low for patients treated with hypothermia [39]. Edgar et al. observed that outcome prediction after CA was affected by sedation and called for more objective predictors that are not affected by drug use or metabolism [40]. In this study, EEG parameters were associated with the neurological function outcome and the prediction power was not affected in predicting survival when the hydrogen treatment data were included. This outcome showed that EEG could be used to assess the efficacy of hydrogen inhalation in real time and provided evidence for adjusting the administration of hydrogen. Prediction of outcome by EEG could also help caregivers make sensible decisions regarding the intensity of care. With its efficacy in neuroprotection and the feasibility of real-time outcome prediction, hydrogen inhalation therapy may be successfully translated into clinical practice.

We recognize several limitations in the current study. First, the female Sprague-Dawley rats were used in this study, and the rate of metabolism and physiology of the small rodent are different from those of humans. Thus, the results of the study cannot be extrapolated to humans. Second, the method of CA induction and the duration of untreated CA are important factors that affect neurological outcome. However, this study evaluated only the quantitative EEG characteristics in the setting of 5 min of untreated asphyxial CA. Additionally, the NDS used to evaluate neurological outcome might be inappropriate if the animals died due to myocardial damage, organ failure, or other acute disease. Third, we observed that inhalation of 2% hydrogen with 98% oxygen for 1 h immediately after ROSC significantly affected the quantitative EEG characteristics, but other concentrations of hydrogen and the duration of hydrogen administration were not tested. Fourth, the comparison of the area under the ROC between the Ctrl and H2 groups was not performed because only 2 animals did not survive to 96 h in the H2 group. Therefore, whether the predictive capacity of the quantitative EEG characteristics is independent of hydrogen treatment still needs to be validated.

## 5. Conclusions

Hydrogen inhalation improved the quantitative EEG characteristics at early postresuscitation in an asphyxial CA rat model. Quantitative EEG indicators during early postresuscitation not only reflected hydrogen inhalation but also provided a reliable prognosis of the outcome.

## Figures and Tables

**Table 1:** Data at baseline, CA induction and resuscitation.

	Ctrl (*N* = 20)	H2 (*N* = 20)	*P*-value
Body weight, g	236.90 ± 24.38	236.50 ± 21.83	0.96
Heart rate, bpm	431.75 ± 31.21	418.25 ± 48.77	0.31
MAP, mmHg	132.25 ± 8.30	131.65 ± 8.41	0.82
Core temperature, °C	36.90 ± 0.15	36.91 ± 0.11	0.78
Asphyxial time to induce CA, sec	164.60 ± 27.79	172.85 ± 30.05	0.37
CPR time, sec	86.55 ± 34.17	78.80 ± 14.61	0.36
Total dose of epinephrine, ug	0.18 ± 0.04	0.18 ± 0.04	0.84
Successful resuscitation, %	100	100	1

Ctrl, control; H2, hydrogen inhalation; MAP, mean arterial pressure; CA, cardiac arrest; CPR, cardiopulmonary resuscitation.

**Table 2:** Neurological deficit score.

	Ctrl (*N* = 20)	H2 (*N* = 20)		*P*-value
At 24 h	315.00 [228.75–475.00]		177.50 [116.25–215.00]	<0.01
At 48 h	490.0 [142.50–500.00]	105.00 [72.50–175.00]		<0.01
At 72 h	500.0 [101.25–500.00]	45.00 [26.25–113.75]		<0.01
At 96 h	500.00 [57.50–500.00]	7.50 [0.00–113.75]		<0.01

Ctrl, control; H2, hydrogen inhalation.

**Table 3:** Arterial blood gas analyses before and after resuscitation.

	BL	PR120	PR240
PH			
Ctrl	7.43 ± 0.04	7.35 ± 0.10	7.41 ± 0.04
H2	7.44 ± 0.06	7.39 ± 0.09	7.42 ± 0.03
PaO_2_, mmHg			
Ctrl	87.35 ± 8.71	93.05 ± 18.18	92.67 ± 10.98
H2	82.00 ± 11.03	95.65 ± 19.40	84.73 ± 12.62
PaCO_2_, mmHg			
Ctrl	38.06 ± 5.57	39.72 ± 10.51	37.36 ± 5.68
H2	36.95 ± 7.23	38.88 ± 14.45	37.75 ± 6.73
SaO_2_, %			
Ctrl	96.85 ± 1.39	93.55 ± 10.62	96.67 ± 2.27
H2	96.20 ± 1.61	96.50 ± 1.67	95.38 ± 2.20

BL, baseline; PR, postresuscitation; Ctrl, control group; H2; hydrogen group.

## Data Availability

The data used to support the findings of this study are included within the article. The datasets analyzed during the current study are available from the corresponding author on reasonable request.
